# Feeding a Multi-Enzyme Blend to Enhance the Nutrient Digestibility of Wheat-Canola Expeller Diets in Ileal-Cannulated Weaned Pigs

**DOI:** 10.3390/ani14111644

**Published:** 2024-05-31

**Authors:** A. Janine Soderstrom, Li Fang Wang, Rob Patterson, Eduardo Beltranena, Ruurd T. Zijlstra

**Affiliations:** 1 Department of Agricultural, Food and Nutritional Science, University of Alberta, Edmonton, AB T6G 2P5, Canadaeduardob@ualberta.ca (E.B.); 2 CBS Bio Platforms, Calgary, AB T2C 0J7, Canada

**Keywords:** canola, coproduct, digestibility, enzyme, pig

## Abstract

**Simple Summary:**

Feeding pigs to convert human non-edible plant coproducts into pork for human nutrition is important for sustainable food production systems. From canola seed, oil is used for cooking or biofuel production, and canola expeller is one of the high-fiber coproducts. Feed enzymes to degrade fiber may enhance nutrient utilization of diets containing high-fiber ingredients in pigs. Pigs were surgically prepared so that, in addition to feces, also digesta could be collected from the end of the small intestine to study the effect of a feed additive containing multiple enzymes on nutrient digestibility. Feeding the multi-enzyme blend increased the nutrient digestibility of the control diet containing wheat and barley as cereal grain, but not the nutrient digestibility of the canola expeller. Thus, research is needed to identify enzyme combinations or feed processing measures that increase the nutrient digestibility of canola expeller.

**Abstract:**

Canola expeller (CE) contains ~200 g/kg residual oil, but also fiber that impairs nutrient digestibility in weaned pigs. To study if feed enzymes increase digestibility, six diets containing either the basal or two CE samples mixed in at 250 g/kg (CE-A or CE-B) were formulated with or without a multi-enzyme blend containing cellulase, xylanase, glucanase, amylase, protease, invertase, and pectinase. The basal diet containing 620 g/kg wheat and 150 g/kg barley served as control. Twelve ileal-cannulated barrows (9–15 kg) were fed the six diets in a replicated 6 (pigs) × 3 (periods) Youden square. Ileal digestibility of gross energy and amino acids was 5% greater for basal than CE diets without differences between CE samples. Diet energy values were 4% greater for CE than basal diets due to residual oil in CE. Inclusion of the multi-enzyme blend increased total tract digestibility of energy of the basal but not CE diets by 2%. Net energy value was greater for CE-A than CE-B because CE-A contained more residual oil. In conclusion, feeding 250 g/kg CE increased diet energy values; thus, CE can substitute added fat in weaned pig diets. Feeding the multi-enzyme blend increased the energy digestibility of wheat and barley-based diets fed to weaned pigs. However, research is needed to identify enzyme combinations that increase the nutrient digestibility of CE.

## 1. Introduction

The expansion of canola production in western Canada has coincided with increased tonnage of seed; however, not all canola seed grown meets human food oil grade. Excess food-grade and lower-grade canola seed can be diverted to feed markets (cake and oil feeding) and biodiesel production [1]. Facilities without solvent extraction capability produce canola coproducts such as ‘cold-pressed’ canola cake (around 200 g/kg remaining oil) or canola expeller (CE; up to 200 g/kg remaining oil) based on the processing equipment used [2,3]. Feeding excess and non food-grade canola seed can increase sustainability of the crop and livestock sectors by achieving circularity [4]. Locally produced CE can be a discounted feedstuff [3,5], and its feeding provides an opportunity to lower feed costs [6,7]. Beyond soybean meal (SBM), canola coproducts are a good source of protein and amino acids (AAs) in swine diets. Because of oil pressing without solvent extraction, CE contains more residual oil (100–200 g oil/kg) than canola meal (CM; 26 g crude fat/kg) and SBM (22 g crude fat/kg), increasing its net energy (NE) value. Apart from differences in lipid content between CE and CM, variation in available nutrients among CE samples may also occur from processing. For example, extruded canola seed can be subjected to seed conditioning and pressing friction temperature variations (95–130 °C) that can lower the bioavailability of some AAs, such as lysine [8,9]. Other factors, e.g., cultivar, seed quality, agronomic conditions, and processing equipment variables, can also influence CE digestibility [10].

Canola coproducts contain several anti-nutritional factors (ANFs). Glucosinolates are low in modern canola cultivars, yet are concentrated in CE after pressing, and could reduce CE and CM digestibility [3,7]. Phytic acid impairs digestibility of phosphorus, minerals, and AAs [11]. Finally, the fiber content of CE is similar to that of CM, but greater than that of dehulled SBM, and can hinder digestibility of energy and other nutrients [12,13]. The CE contains 240 g NDF/kg and 160 g ADF/kg, compared with 82 and 53 g/kg in dehulled SBM, respectively [14,15]. The fiber contained in canola coproducts is rigid and complex and primarily limits their inclusion rate in weaned pig diets, as glucosinolate content is no longer an issue (<10 μmol/kg) [16]. Dietary inclusion of feed enzymes may increase the digestibility of fiber in canola coproducts. Recently, supplementation of a multi-enzyme blend to degrade complex fiber structures, including xylanase, glucanase, cellulase, mannanase, invertase, protease, pectinase, and amylase, increased the in vitro dry matter (DM) digestibility of canola coproducts [17]. The degradation of fiber in CE may increase the efficacy of gastric and pancreatic enzymes to digest dietary nutrients and thereby allow young pigs to meet their high AA and NE requirements [18,19]. Finally, the ileal nutrient digestibility of CE in weaned pigs has not been described often. More reports are needed to convince pig producers of the benefits of feeding canola coproducts.

The null hypotheses of the present study were that diet nutrient and energy digestibility would not change with inclusion of CE, that the nutritional value would not differ between two CE samples, and that the addition of a multi-enzyme blend would not alter the nutrient digestibility of CE diets or CE individually. The objectives were to determine and compare the coefficients of apparent ileal digestibility (CAID) and coefficients of apparent total tract digestibility (CATTD) of energy and nutrients in a basal diet and two diets containing 250 g/kg of different CE samples with or without the inclusion of a multi-enzyme blend fed to ileal-cannulated weaned pigs.

## 2. Materials and Methods

The animal experiment was conducted at the Swine Research and Technology Centre of the University of Alberta (Edmonton, AB, Canada) with animals provided from its swine herd following animal use approval and review of procedures by the Animal Care and Use Committee for Livestock following guidelines established by the Canadian Council on Animal Care [20].

### 2.1. Test Materials and Diet Processing

A wheat-barley basal diet was sourced from Country Junction Feeds (Wetaskiwin, AB, Canada). The basal diet was formulated based on NE values from NRC [15] and digestible amino acid values from INRA [21]. Two CE samples were sourced locally: canola expeller A (CE-A) from Milford Hutterite Colony (Raymond, AB, Canada) and canola expeller B (CE-B) from Prairie Home Hutterite Colony (Conrad, AB, Canada). Canola expeller diets included 750 g basal/kg plus 250 g CE-A or CE-B/kg, with or without 0.5 g/kg of multi-enzyme blend. The multi-enzyme blend contained, per gram, 900 U cellulase, 1200 U xylanase, 250 U glucanase, 12,000 U amylase, 6000 U protease, 700 U invertase, and 2400 U pectinase (Superzyme-Conc^®^, CBS Bio-Platforms; Calgary, AB, Canada). Diets included 5 g titanium dioxide (TiO_2_)/kg as an indigestible marker for digestibility determination (Table 1). Diets were mixed (6 min) using a 300 kg horizontal paddle mixer (model 3061; Marion Process Solutions, Marion, IA, USA) and were fed as mash. Particle size of the ingredients and diets was analyzed using a mechanical sieve shaker (Model RX-29, W.S. Tyler, St. Catherines, ON, Canada) following the method of the American Society of Agricultural and Biological Engineers (Table 2) [22].

### 2.2. Experimental Design and Management

This experiment was designed as a replicated 6 (pigs) × 3 (periods) Youden square to reach 6 observations per dietary treatment. In total, 12 barrows (Duroc × Large white/Landrace F1; 12 to 18 kg body weight) were surgically cannulated at the distal ileum. Pigs recovered from cannulation surgery and had a gradual increase in feed allowance over 7 days. Introduction of the experimental diets was carried out by substituting 250, 500, and 750 g/kg of the pre-surgical diet with the specific experimental diet over 3 days, ending with 1000 g/kg experimental diet by the beginning of the first diet acclimation period. Daily feed allowance was calculated at 3.0 (group 1) or 2.8 (group 2) × maintenance digestible energy (DE; 110 kcal of DE per kg of BW^0.75^) [23] divided into two meals offered at 08:00 and 15:00. Throughout the trial, pigs were housed in individual metabolic pens measuring 1.2 m length × 1.2 m width × 0.95 m height (1.8 m^2^). The pens had walls made of polyvinyl plastic planking with 0.16 m^2^ plexiglass windows on three sides. Free access to water was provided from a cup drinker placed 0.25 m above the floor beside the feeder. The stainless-steel feeder measured 0.35 m width × 0.4 m height with the trough 0.12 m above the pen floor. The climate-controlled room was maintained at 25 ± 2.5 °C for pigs < 15 kg and 22 ± 2.5 °C for pigs > 15 kg. Lighting was provided daily from 07:00 to 19:00.

Each 9-day period started with 5 days of acclimation to the experimental diet, followed subsequently by 2-day collection of feces and 2-day collection of digesta. Feces were collected with plastic bags secured between leather and Velcro rings glued around the tail and anus for 48 h starting at 08:00 and monitored throughout both fecal collection days [24]. Feces from each pig were promptly pooled and frozen. Following completion of collection, feces were thawed, homogenized and subsampled. Digesta was collected continuously from 08:00 to approximately 19:00 through the opened T-cannula via attached plastic collection bags containing 15 mL of 50 g/kg formic acid to prevent bacterial fermentation. Meals were provided at 08:00, 10:00, 13:00, and 15:00 during digesta collection to allow for a continuous flow of digesta from the cannula. Bags containing digesta were promptly pooled and frozen. Following completion of collection, digesta was thawed, homogenized, and subsampled. Once subsampled, feces and digesta specimens remained frozen (−20 °C) until lyophilization.

### 2.3. Chemical Analyses

Test ingredients, diets and lyophilized digesta and feces were ground through a 1 mm screen using a centrifugal mill (Model ZM200, Retch GmbH, Haan, Germany). Test ingredients and diets were analyzed for starch (assay kit STA-20; method 76-13) [25] and for crude fat using AOAC methods (method 920.39A), neutral detergent fiber (NDF) assayed without a heat-stable amylase and expressed inclusive of residual ash [26], acid detergent fiber (ADF) inclusive of residual ash (method 973.18) and ash (method 942.05) at the Agricultural Experiment Station Chemical Laboratories (ESCL), University of Missouri (Columbia, MO, USA) [27]). Test ingredients were analyzed for total dietary fiber (method 985.29), soluble and insoluble dietary fiber (method 991.43), crude fiber (method 934.01), calcium (method 968.08), phosphorus (method 946.06), amino acids (AAs; method 982.30E a-c), and chemically available lysine (method 975.44) at ESCL [27]. Digesta was analyzed for moisture, crude protein (CP), and AAs at ESCL. Diets, digesta, and feces were analyzed for DM (method 930.15; AOAC, 2006), CP by LECO (nitrogen × 6.25; method 990.03), gross energy (GE) using an adiabatic bomb calorimeter (model 5003; Ika-Werke, Staufen, Germany) and titanium dioxide (TiO_2_) content at the University of Alberta [28].

### 2.4. Calculations

The CAID (AA) and CATTD of DM, CP, and GE of the diets were calculated for each diet using the index method via the following calculation:

$$CAID\;or\;CATTD=1-\left(\frac{\left(Concentration\;of\;TiO_{2_{feed}}\right)\times \left({Concentration\;of\;component}_{digesta\;or\;feces}\right)}{\left(Concentration\;of\;TiO_{2_{digesta\;or\;feces}}\right)\times ({Concentration\;of\;component}_{feed})}\right)$$

The CAID and CATTD of DM, CP, AAs, and GE of the two CE ingredients were calculated using the difference method via the following calculation [29]:

$$CAID\;or\;CATTD=\left(\frac{\left({CAID\;or\;CATTD}_{test\;diet}\times proportion_{test\;diet}\right)-\left({CAID\;or\;CATTD}_{basal}\times proportion_{basal}\right)}{proportion_{test\;ingredient}}\right)$$

Diet and ingredient DE (MJ/kg) was calculated by the following formula:

$$DE=\left(GE_{Diet\;or\;ingredient}\times GE\;{CAID\;or\;CATTD}_{diet\;or\;ingredient}\right)$$

Diet and ingredient NE (kcal/kg) was calculated using the equation of Noblet et al. [30] as adopted by the NRC [15], where nutrient values are expressed as g/kg DM, then converted to MJ/kg:

$$NE=\left(0.7\times DE\right)+\left(1.61\times EE\right)+\left(0.48\times Starch\right)-\left(0.91\times CP\right)-(0.87\times ADF)$$

### 2.5. Statistical Analyses

Data were analyzed using the GLIMMIX procedure of SAS (Version 9.4; SAS Institute Inc., Cary, NC, USA) as a replicated 2 × 3 (Diets) and 2 × 2 (Ingredients) factorial arrangement. Normality and homogeneity of variance for the residual of each variable and carry-over effect of diets fed in the previous period were tested prior to ANOVA analysis. Pig was the experimental unit. Diet was the fixed effect, whereas square, pig nested within square and period were random terms. For multiple comparisons, *p* values were adjusted using the Tukey–Kramer method. To test the hypothesis, *p* < 0.05 was considered significant.

## 3. Results

Pigs remained healthy throughout the experiment and maintained daily feed intake regardless of the test diet offered. Diets including CE contained 13% less starch and 10% less ash than the basal diets (Table 2). Diets including CE contained more CP, crude fat, ADF, and NDF and 1.35 MJ/kg more GE than the basal diets.

The CE-A contained 26 g/kg more crude fat than CE-B (Table 3). However, CE-B contained 12 g more CP/kg and 5 g more total dietary fiber (TDF)/kg, and had a 49 μm larger particle size than that of CE-A. For both CE samples, 98% of the TDF was insoluble.

For CATTD of DM and GE and diet NE values, an interaction between diet and enzyme inclusion was observed (*p* < 0.05; Table 4). Dietary inclusion of CE reduced (*p* < 0.05) the diet CAID and CATTD of DM, GE, and CP for both diets with and without enzyme. Dietary inclusion of CE increased (*p* < 0.05) diet ileal and total tract DE and NE values. Inclusion of the multi-enzyme blend increased (*p* < 0.05) the CATTD of DM and GE of the basal diet by 0.012 and 0.015, respectively. Inclusion of the multi-enzyme blend increased the NE value of the basal diet by 0.34 MJ/kg.

Dietary inclusion of CE decreased (*p* < 0.05; Table 5) the diet CAID of AAs. For the CAID of diet methionine, tryptophan, and tyrosine, an interaction between diet and enzyme inclusion was observed (*p* < 0.05). Specifically, dietary enzyme inclusion reduced (*p* < 0.05) the CAID of tryptophan for CE-A, reduced (*p* < 0.05) the CAID of methionine for CE-B and increased (*p* < 0.05) the CAID of tryptophan and tyrosine for CE-B.

The CAID of DM, GE, and CP and the CATTD of DM and GE did not differ between the two CE samples (Table 6). Ingredient NE value was 0.87 MJ/kg lower (*p* < 0.05) for CE-B than for CE-A. Dietary enzyme increased (*p* < 0.05) the CATTD of CP for CE-A only.

The CAID of most AAs did not differ between CE-A and CE-B (Table 7). However, inclusion of dietary enzyme decreased (*p* < 0.05) the CAID of methionine for CE-B only.

## 4. Discussion

### 4.1. Canola Expeller Inclusion

The CE may contain up to 200 g remaining oil/kg instead of the 30 g crude fat/kg in canola meal; thus, inclusion in weaned pig diets can increase dietary energy value [31,32]. The two CE samples obtained for the present study contained 66–93 g/kg more crude fat than previously reported for CE [14,31], confirming that nutritional quality and energy values differ among CE samples. Both CE samples fed contained ADF similar to previously reported values [31]. In the present study, a highly digestible wheat and barley-based basal diet was fed as a control. Adding 250 g CE/kg decreased the diet CAID and CATTD of DM, GE, and CP and CAID of total AAs, likely because of increased dietary fiber content, as reported in previous studies [14,31,32]. Non-starch polysaccharides present in the CE diets likely reduced the digestibility of other nutrients, particularly in weaned pigs because of an underdeveloped hindgut [33]. In previous nutrient digestibility studies feeding ileal-cannulated weaned pigs, dietary inclusion of 100 g CM/kg reduced the CAID of CP and AAs [34]. As the first limiting AA, the CAID of lysine is often considered more important than CP digestibility [3]. In the present study, the CAID of lysine was reduced compared with the control, a reduction consistent with that reported by Mariscal-Landín et al. in weaned pigs [34]. Pressing canola seed can create high temperatures in the product through steam conditioning prior to flaking and friction within the barrel of the expeller press. However in the present study, 97% of the lysine was chemically available in both CE samples, indicating that Maillard reactions were biologically not relevant and heat damage during processing was minimal [14]. In the present study, the remaining oil in the CE samples increased diet DE and NE, ileal DE and ileal NE values compared with feeding the control diet (Table 4). This approach contrasts similar studies that formulate consistent NE values for the experimental diets [3,35]. Increasing the inclusion rate up to 250 g CE/kg in weaned pig diets may lower the required inclusion of energy-rich fats to maintain diet energy value and may thereby lower feed cost.

The nutritional value of CE may vary among small-scale domestic processing facilities in Canada because of canola seed quality and processing variables [16]. Regarding seed, the cultivar, growing conditions, and harvest and storage conditions are important factors. Regarding processing, the initial moisture content, press type, and processing temperature are important. Among 11 canola solvent extraction processing facilities in western Canada, CM varied in nutrient content [16]. Specifically, for CM, it ranged from 402–429 g/kg in CP and from 26.0–43.0 g/kg in DM in ether extract (EE). Interestingly, without differences in NSP content, NDF and lignin content did differ among CM samples, indicating variation in nutritional value. In the present study, the available lysine content was consistent between the two CE samples, indicating similar temperature conditions during processing. These two CE samples differed mainly in NDF, ADF, and crude fat content and particle size (Table 3). The two CE samples did not differ in the CAID and CATTD of DM, GE, and CP, likely because their fiber content and physiochemical properties were insufficiently different to affect nutrient digestibility. However, CE-A had a greater NE value than CE-B, likely because CE-A contained more crude fat [36]. Fat in the diet may decrease the passage rate and thereby increase nutrient digestion and absorption [37]. For the diet NE value increased by including CE, the effect of increased DE value due to the remaining oil was greater than the reduced CATTD of energy due to increased dietary fiber and fat content [36].

### 4.2. Enzyme Inclusion

Enzyme inclusion in pig diets has been studied extensively to increase nutrient digestibility and subsequent growth performance in all stages of production [18]. Weaned pig diets provide the largest enzyme market in swine production because several substrates such as NSP, protein, and phytate can reduce nutrient digestibility and exacerbate the post-weaning lag or increase incidence of diarrhea partly due to an immature gut [38]. With increased diet complexity and fibrous ingredients, diets can be supplemented with multi-enzyme blends that can be added to feed as a single additive to increase the degradation of dietary substrates that hinder nutrient digestion [39,40,41]. Enzyme blends can have an additive or synergistic effect to increase nutrient digestibility in pig diets further than can be achieved by feeding a single enzyme [41]. Dietary inclusion of single or multiple enzymes increased ileal digestibility of GE, DM, and CP with xylanase and phospholipase in wheat-based diets [39] and with cellulase, galactanase, mannanase, and pectinase in corn-based diets fed to weaned pigs [42]. In the present study, inclusion of the multi-enzyme blend increased the CATTD of DM and GE for the wheat and barley-based basal diet. The enzyme blend in the present experiment included cellulase, xylanase, glucanase, amylase, invertase, and pectinase activities that were consistent with the substrates present in the basal diet, specifically arabinoxylans in wheat and β-glucans in barley. Interestingly, although protease was included in the enzyme blend, neither diet CAID of AAs nor CATTD of CP were increased in the present study. However, looking at the ingredients independently, enzyme inclusion increased the CATTD of CP solely for CE-A. In studies with a similar enzyme blend containing protease, the CAID or coefficient of standardized ileal digestibility (CSID) of CP or AAs was not affected in growing pigs fed fibrous camelina cake [19]. Particle size is another consideration for enzyme efficacy. Increased particle size (>800 μm) typically leads to lower nutrient digestibility but may result in greater enzyme efficacy [43]. In the present study, the particle size of the CE diets was consistent with that of the controls and the 600 μm industry standard for wheat-based mash diets, indicating that particle size was not a factor for the lack of enzyme efficacy in the CE diets (Table 2) [44].

In the present study, feeding the enzyme blend did not affect the nutrient digestibility of diets including either CE. Similarly, the enzyme blend did not increase the CAID of DM, GE, and CP or the CATTD of DM and GE of the CE samples individually. Canola seed itself is small in size (measuring approximately 1 mm in diameter) compared with cereal grains (e.g., 2.75 mm for wheat) or pulse grains (e.g., 6 mm for field pea) [45,46]. Because of this small size, the highly ligneous hull (300 g/kg of CM) can severely reduce enzyme efficacy and overall nutrient digestibility [47]. Made up primarily of insoluble fiber, canola hull and therefore expeller are difficult to be degraded enzymatically through decreased contact and an increased passage rate [40,48]. Often, the effects of multi-enzyme inclusion are therefore inconsistent in pig diets containing canola coproduct and other insoluble fiber-rich ingredients. Similarly to the present study, the inclusion of a multi-enzyme blend did not affect energy digestibility in diets containing cassava meal fed to nursery pigs [49]. Similarly, supplementation of xylanase and β-glucanase blend did not affect the CAID of DM, GE, ADF, and NDF in weaned pigs fed 250 g CM/kg [50]. Unlike the present trial, multi-enzyme inclusion did increase the CAID of DM, GE, CP, and starch in diets containing 60 g CM/kg [42] and did increase the CAID of DM, GE, and CP in diets containing 200 g rice bran/kg fed to weaned pigs [51].

### 4.3. Canola Expeller and Enzyme Interaction

Inclusion of the multi-enzyme blend increased the CATTD of DM and GE for the basal diet only, indicating the importance of matching enzyme to the type of substrate. In diets containing CE, the presence of lignin might have reduced the efficacy of cellulase, lowering the overall enzyme activity compared with feeding the wheat-based diet alone [52]. However, in an in vitro study, similar enzyme blends containing xylanase, glucanase, cellulase, mannanase, invertase, protease, amylase and pectinase added to similar substrates (cold-pressed canola cake) increased the digestion of nutrients [17]. Previous reports attest to the accuracy of using in vitro digestibility results to predict enzyme efficacy in swine [53]. In vivo digestibility is subject to more factors that can affect digestibility coefficients versus in vitro digestibility. Pig factors such as feed and water intake and passage rate, environmental temperature, and sample collection can interfere with the measured values for nutrient digestion, potentially explaining the reduced enzyme efficacy in CE observed in the present study. In growing pigs, feeding multi-enzyme blends increased the metabolic energy value, CSID of CP and AAs, and CATTD of ADF and NDF in diets containing double-low rapeseed expeller [54]. The dose of enzyme supplemented to the diet is critical for ensuring adequate substrate hydrolysis. Increasing the enzyme dose has been associated with a linear increase and subsequent plateau in nutrient digestibility and pig performance [38,50,55]. With more substrate for the supplemented enzymes present in the CE than in control diets in the present trial, the enzyme dose might have been insufficient for proper substrate hydrolysis. Similarly to the present trial, dietary inclusion of 250 g CM/kg reduced the CATTD of DM, GE, and CP in weaned pigs; however, feeding a multi-carbohydrase enzyme blend increased the CATTD of DM, GE, and CP compared with a control using SBM [56]. In the present trial, the enzyme increased the NE value of the basal diet by 0.34 MJ/kg. Despite this increase, diet NE, diet DE, and ileal DE values were still greater for CE diets regardless of enzyme inclusion. Our results indicate that the enzyme blend composition and dose still need to be elucidated for weaned pig diets containing CE.

## 5. Conclusions

The inclusion of 250 g CE/kg in weaned pig diets reduced the CAID and CATTD of GE, CP and AAs. Although the fiber in CE reduced nutrient digestibility, the extra energy that CE provided to the diet from remaining oil increased the NE, DE and ileal DE values of the experimental diets. Slight variations in energy and CP digestibility between the two CE samples indicated that variation in nutritional value exists among CE sources and should be considered in diet formulations. Multi-enzyme blend inclusion increased nutrient digestibility solely in the basal diet, indicating a proper match between the enzyme blend and substrate for the wheat and barley-based diets. However, the proper enzyme blend composition and dose still need to be elucidated for diets including canola expeller.

## Figures and Tables

**Table 1:** Ingredient composition (g/kg diet, as fed) of experimental diets.

Ingredient	Without Enzyme				Multi-Enzyme		
	Basal	CE-A	CE-B		Basal	CE-A	CE-B
Wheat	615.98	461.98	461.98		615.67	461.67	461.67
Hulled barley	150.60	112.94	112.94		150.52	112.87	112.87
Canola expeller A (CE-A) ^1^	–	250.00	–		–	250.00	–
Canola expeller B (CE-B) ^2^	–	–	250.00		–	–	250.00
Menhaden fish meal	99.30	74.48	74.48		99.25	74.43	74.43
Soy protein concentrate HP300 ^3^	99.30	74.48	74.48		99.25	74.43	74.43
Limestone	10.90	8.18	8.18		10.89	8.17	8.17
Mono/di-calcium phosphate	7.95	5.96	5.96		7.95	5.96	5.96
Salt	5.96	4.47	4.47		5.96	4.47	4.47
Choline chloride 600 g/kg	0.99	0.74	0.74		0.99	0.74	0.74
Mineral premix ^4^	1.88	1.41	1.41		1.88	1.41	1.41
Vitamin premix ^5^	0.54	0.41	0.41		0.54	0.40	0.40
TiO_2_	6.60	4.95	4.95		6.60	4.95	4.95
Multi-enzyme blend ^6^	–	–	–		0.50	0.50	0.50

^1^ Milford Hutterite Colony, Raymond, AB, Canada. ^2^ Prairie Home Hutterite Colony, Conrad, AB, Canada. ^3^ HP300 (Hamlet Protein Inc., Findlay, OH, USA). ^4^ Supplied per kilogram of basal diet: 532 mg Zn, 126 mg Cu, 527 mg Fe, 103 mg Mn, 1.0 mg I, 0.63 mg Se. ^5^ Supplied per kg of basal diet: 12,000 IU vitamin A, 1200 IU vitamin D, 111 IU vitamin E, 104 mg niacin, 28 mg pantothenic acid, 2.6 mg folacin, 15.8 mg riboflavin, 8.7 mg pyridoxine, 8.2 mg thiamine, 520 mg choline, 4.8 mg vitamin K, 0.76 mg biotin, 0.04 mg vitamin B_12_. ^6^ Supplied (U/kg of diet): cellulase, 450; xylanase, 600; glucanase, 125; amylase, 6000; protease, 3000; invertase, 350; pectinase, 1200 (Superzyme-Conc^®^; CBS Bio-Platforms, Calgary, AB, Canada). Xylanase was analyzed as a marker in diets to confirm proper multi-enzyme blend addition: intrinsic xylanase averaged 97 Y/kg in diets without enzyme blend and averaged 850 U/kg in diets with enzyme blend.

**Table 2:** Analyzed nutrient composition (g/kg, as fed) and gross energy (GE) value of experimental diets.

Nutrient	Without Enzyme				Multi-Enzyme		
	Basal	CE-A	CE-B		Basal	CE-A	CE-B
Moisture	109	109	104		110	105	104
Starch	255	225	220		307	222	198
Crude protein	229	233	241		216	240	241
Neutral detergent fiber	107	130	127		106	126	157
Acid detergent fiber	43.0	56.0	62.8		32.9	62.1	61.3
Ash	73.8	66.4	66.6		73.9	65.7	65.6
Crude fat	9.44	58.6	53.3		11.2	56.7	54.2
Indispensable amino acids							
Arginine	1.19	1.31	1.31		1.22	1.30	1.34
Histidine	0.49	0.56	0.57		0.51	0.56	0.57
Isoleucine	0.88	0.95	0.95		0.90	0.96	0.97
Leucine	1.50	1.62	1.63		1.53	1.64	1.66
Lysine	1.11	1.25	1.23		1.13	1.24	1.25
Methionine	0.39	0.43	0.43		0.39	0.46	0.44
Phenylalanine	0.99	1.03	1.04		1.02	1.06	1.05
Threonine	0.73	0.85	0.88		0.77	0.85	0.87
Tryptophan	0.29	0.28	0.25		0.27	0.23	0.29
Valine	0.98	1.13	1.13		1.02	1.11	1.14
Dispensable amino acids							
Alanine	0.99	1.05	1.07		1.02	1.05	1.08
Aspartic acid	1.69	1.74	1.74		1.71	1.76	1.77
Cysteine	0.34	0.46	0.46		0.37	0.47	0.47
Glutamic acid	4.56	4.59	4.70		4.67	4.64	4.70
Glycine	1.11	1.17	1.20		1.15	1.17	1.20
Proline	1.55	1.55	1.60		1.60	1.65	1.61
Serine	0.79	0.85	0.86		0.81	0.85	0.87
Tyrosine	0.65	0.67	0.62		0.65	0.72	0.68
Total amino acids	20.72	22.00	22.18		21.24	22.22	22.47
GE (MJ/kg)	16.1	17.5	17.4		16.0	17.5	17.4
Xylanase units/kg	112	76	76		814	623	1047
Particle size, µm ^1^							
Mean	578	582	607		559	607	613
Standard deviation	2.13	2.17	2.12		2.30	2.11	2.09

^1^ Particle size was measured in triplicate.

**Table 3:** Analyzed nutrient composition (g/kg, as fed), energy value and particle size of canola expeller samples.

Nutrient	CE-A ^1^	CE-B ^2^
Moisture	85.6	86.3
Crude protein (N × 6.25)	304	316
Total dietary fiber	280	285
Insoluble dietary fiber	274	281
Soluble dietary fiber	5.67	3.7
Neutral detergent fiber	188	194
Acid detergent fiber	139	156
Crude fat	196	172
Starch	55.3	52.4
Ash	51.6	52.7
Phosphorus	8.63	8.6
Calcium	5.92	5.4
Indispensable amino acids		
Arginine	17.6	19.1
Histidine	8.0	8.4
Isoleucine	12.0	13.5
Leucine	20.0	22.3
Lysine	17.3	18.2
Methionine	6.0	6.4
Phenylalanine	12.0	13.2
Threonine	12.0	13.1
Tryptophan	4.0	4.7
Valine	15.2	16.7
Dispensable amino acids		
Alanine	12.7	13.8
Aspartic acid	20	22.1
Cystine	8	8.8
Glutamic acid	49	53.5
Glycine	14.2	15.4
Proline	19	20.6
Serine	11	11.4
Tyrosine	9	9.4
Total amino acids	274.3	297.0
Chemically available lysine	16.7	17.6
GE (MJ/kg)	21.8	21.3
Particle size, µm ^3^		
Mean	676	725
Standard deviation	1.83	1.89

^1^ Milford Hutterite Colony, Raymond, AB, Canada. ^2^ Prairie Home Hutterite Colony, Conrad, AB, Canada. ^3^ Standard deviations based on triplicates.

**Table 4:** Coefficient of apparent ileal digestibility (CAID) and coefficient of apparent total tract digestibility (CATTD) of dry matter, gross energy and crude protein, and the digestible energy (DE) and calculated net energy (NE) values of experimental diets (values standardized to 900 g dry matter/kg) ^1^.

Variable	Without Enzyme				Multi-Enzyme			SEM ^2^	*p*-Value		
	Basal	CE-A	CE-B		Basal	CE-A	CE-B		Diet	Enzyme	Enzyme × Diet
CAID											
Dry matter ^3^	0.737	0.697	0.695		0.747	0.699	0.710	0.010	<0.001	0.133	0.678
Gross energy ^3^	0.757	0.717	0.716		0.764	0.717	0.728	0.010	<0.001	0.252	0.726
Crude protein ^3^	0.827	0.782	0.779		0.815	0.779	0.790	0.008	<0.001	0.752	0.156
CATTD											
Dry matter	0.827 ^b^	0.802 ^c^	0.809 ^c^		0.839 ^a^	0.811 ^c^	0.806 ^c^	0.003	<0.001	0.005	0.019
Gross energy	0.828 ^b^	0.795 ^c^	0.804 ^c^		0.843 ^a^	0.803 ^c^	0.801 ^c^	0.005	<0.001	0.014	0.045
Crude protein ^3^	0.867	0.823	0.841		0.870	0.844	0.842	0.008	<0.001	0.102	0.145
Energy values, MJ/kg											
Diet DE	13.4 ^b^	14.0 ^a^	14.1 ^a^		13.7 ^b^	14.1 ^a^	14.0 ^a^	0.001	<0.001	0.036	0.037
Diet NE ^4^	9.02 ^d^	9.62 ^ab^	9.60 ^ab^		9.36 ^c^	9.67 ^a^	9.46 ^bc^	0.001	<0.001	0.014	<0.001
Diet ileal DE ^5^	12.3	12.6	12.5		12.4	12.6	12.7	0.002	0.024	0.316	0.772
Diet ileal NE ^5^	8.11	8.66	8.53		8.45	8.61	8.57	0.001	<0.001	0.097	0.054

^1^ Least square means based on 6 pig observations per diet. ^2^ SEM = standard error of the mean. ^3^ Basal > CE-A and CE-B (*p* < 0.05); CE-A not different from CE-B. ^4^ Diet NE values were calculated using Equation (5) from Noblet et al. [30] using measured diet DE value and analyzed dietary crude fat, starch, crude protein, and acid-detergent fiber content. ^5^ Basal < CE-A and CE-B (*p* < 0.05); CE-A not different from CE-B. ^a–c^ Within a row, means without a common superscript differ (*p* < 0.05).

**Table 5:** Coefficient of apparent ileal digestibility of amino acids of experimental diets ^1^.

Variable	Without Enzyme				Multi-Enzyme			SEM ^2^	*p*-Value		
	Basal	CE-A	CE-B		Basal	CE-A	CE-B		Diet	Enzyme	Enzyme × Diet
Indispensable amino acids											
Arginine ^3^	0.895	0.864	0.860		0.897	0.861	0.868	0.006	<0.001	0.549	0.382
Histidine ^3^	0.863	0.841	0.839		0.869	0.840	0.848	0.007	<0.001	0.298	0.544
Isoleucine ^3^	0.866	0.811	0.804		0.865	0.812	0.817	0.007	<0.001	0.352	0.351
Leucine ^3^	0.869	0.825	0.819		0.869	0.826	0.832	0.007	<0.001	0.270	0.391
Lysine ^3^	0.836	0.810	0.794		0.836	0.798	0.811	0.010	<0.001	0.748	0.145
Methionine	0.888 ^a^	0.854 ^a^	0.851 ^a^		0.886 ^a^	0.863 ^a^	0.805 ^b^	0.012	<0.001	0.066	0.009
Phenylalanine ^3^	0.873	0.830	0.825		0.874	0.832	0.837	0.007	<0.001	0.200	0.500
Threonine ^3^	0.804	0.759	0.751		0.806	0.746	0.760	0.009	<0.001	0.877	0.223
Tryptophan	0.874 ^a^	0.835 ^b^	0.801 ^c^		0.862 ^a^	0.791 ^c^	0.833 ^b^	0.009	<0.001	0.121	<0.001
Valine ^3^	0.840	0.790	0.777		0.840	0.786	0.792	0.008	<0.001	0.619	0.143
Dispensable amino acids											
Alanine ^3^	0.825	0.796	0.786		0.825	0.788	0.799	0.009	<0.001	0.767	0.309
Aspartic acid ^3^	0.809	0.774	0.760		0.804	0.766	0.776	0.009	<0.001	0.799	0.129
Cystine ^4^	0.777	0.768	0.755		0.786	0.749	0.776	0.012	0.037	0.625	0.069
Glutamic acid ^3^	0.904	0.877	0.875		0.913	0.875	0.885	0.006	<0.001	0.122	0.346
Glycine ^3^	0.778	0.744	0.743		0.787	0.719	0.755	0.017	<0.001	0.924	0.232
Proline ^3^	0.865	0.825	0.829		0.880	0.833	0.836	0.008	<0.001	0.045	0.807
Serine ^3^	0.828	0.786	0.777		0.826	0.776	0.785	0.008	<0.001	0.734	0.266
Tyrosine	0.880 ^a^	0.830 ^bc^	0.805 ^c^		0.872 ^a^	0.837 ^b^	0.837 ^b^	0.008	<0.001	0.029	0.009
Total amino acids ^3^	0.855	0.818	0.811		0.859	0.813	0.823	0.007	<0.001	0.410	0.274

^1^ Least square means based on 6 pig observations per diet. ^2^ SEM = standard error of the mean. ^3^ Basal > CE-A and CE-B (*p* < 0.05); CE-A not different from CE-B. ^4^ Basal > CE-A (*p* < 0.05); CE-A not different from CE-B. ^a–c^ Within a row, means without a common superscript differ (*p* < 0.05).

**Table 6:** Coefficient of apparent ileal and coefficient of total tract digestibility of dry matter, gross energy, and crude protein, and digestible energy and calculated net energy values of canola expeller samples (values standardized to 900 g dry matter/kg) ^1^.

Variable	Without Enzyme			Multi-Enzyme		SEM ^2^	*p*-Value		
	CE-A	CE-B		CE-A	CE-B		CE	Enzyme	Enzyme × CE
CAID ^3^									
Dry matter	0.586	0.554		0.552	0.597	0.042	0.825	0.812	0.099
Gross energy	0.632	0.608		0.609	0.644	0.024	0.791	0.699	0.093
Crude protein	0.678	0.673		0.696	0.735	0.029	0.420	0.081	0.320
CATTD ^3^									
Dry matter	0.731	0.748		0.732	0.710	0.017	0.883	0.124	0.097
Gross energy	0.724	0.736		0.721	0.699	0.018	0.766	0.104	0.147
Crude protein	0.719 ^b^	0.771 ^ab^		0.794 ^a^	0.781 ^ab^	0.022	0.355	0.007	0.024
Energy value MJ/kg									
DE	15.5	15.4		15.4	14.6	0.004	0.228	0.104	0.147
NE ^4^	10.6	10.3		10.6	9.73	0.003	0.023	0.104	0.147
Ileal DE	13.5	12.8		13.0	13.5	0.005	0.739	0.707	0.095
Ileal NE	9.26	8.41		8.91	8.93	0.004	0.206	0.707	0.095

^1^ Least square means based on 6 pig observations per diet. ^2^ SEM = standard error of the mean. ^3^ Digestibility coefficients of dry matter, crude protein and gross energy in canola expeller were calculated using the difference method [29], where the basal diet without enzyme was used as basal diet for the test ingredients CE-A and CE-B without enzyme, and the basal diet with enzyme was used as basal diet for the test ingredients of CE-A and CE-B with enzyme. ^4^ Ingredient NE values were calculated using the calculated ingredient DE value and analyzed ingredient crude fat, starch, crude protein, and acid-detergent fiber content using Equation (5) from Noblet et al. [30]. ^a,b^ Within a row, means without a common superscript differ (*p* < 0.05).

**Table 7:** Coefficient of apparent ileal digestibility of amino acids of canola expeller samples ^1,2^.

Variable	Without Enzyme			Multi-Enzyme		SEM ^3^	*p*-Value		
	CE-A	CE-B		CE-A	CE-B		CE	Enzyme	Enzyme × CE
Indispensable amino acids									
Arginine	0.802	0.800		0.789	0.818	0.014	0.196	0.770	0.138
Histidine	0.801	0.801		0.782	0.810	0.017	0.269	0.672	0.255
Isoleucine	0.692	0.684		0.694	0.721	0.021	0.506	0.206	0.256
Leucine	0.727	0.722		0.727	0.755	0.018	0.398	0.218	0.225
Lysine	0.760	0.718		0.725	0.766	0.030	0.982	0.774	0.067
Methionine	0.789 ^a^	0.785 ^a^		0.819 ^a^	0.655 ^b^	0.038	0.008	0.086	0.010
Phenylalanine	0.726	0.722		0.727	0.751	0.018	0.456	0.251	0.298
Threonine	0.679	0.665		0.635	0.679	0.027	0.451	0.448	0.116
Tryptophan	0.753 ^a^	0.667 ^b^		0.650 ^b^	0.782 ^a^	0.028	0.259	0.770	<0.001
Valine	0.695	0.673		0.665	0.706	0.021	0.542	0.910	0.052
Dispensable amino acids									
Alanine	0.556	0.565		0.463	0.599	0.060	0.113	0.489	0.162
Aspartic acid	0.688	0.651		0.668	0.711	0.027	0.889	0.313	0.058
Cysteine	0.704	0.703		0.631	0.717	0.041	0.168	0.321	0.159
Glutamic acid	0.458 ^a^	0.456 ^ab^		0.382 ^b^	0.448 ^ab^	0.025	0.093	0.032	0.076
Glycine	0.666	0.667		0.554	0.684	0.060	0.152	0.228	0.159
Proline	0.727	0.748		0.716	0.732	0.022	0.264	0.427	0.879
Serine	0.695	0.672		0.664	0.696	0.021	0.753	0.818	0.090
Tyrosine	0.721 ^ab^	0.655 ^b^		0.761 ^a^	0.766 ^a^	0.023	0.084	<0.001	0.049
Total amino acids	0.734	0.714		0.717	0.751	0.021	0.631	0.512	0.090

^1^ Digestibility coefficients of dispensable and indispensable amino acids in canola expeller were calculated using the difference method [29], where the basal diet without enzyme was used as basal diet for the test ingredients CE-A and CE-B without enzyme, and the basal diet with enzyme was used as basal diet for the test ingredients CE-A and CE-B with enzyme. ^2^ Least square means based on 6 pig observations per diet. ^3^ SEM = standard error of the mean. ^a,b^ Within a row, means without a common superscript differ (*p* < 0.05).

## Data Availability

The data presented in this study are available on request from the corresponding author.
